# Influence of BOLD Contributions to Diffusion fMRI Activation of the Visual Cortex

**DOI:** 10.3389/fnins.2016.00279

**Published:** 2016-06-28

**Authors:** Rebecca J. Williams, David C. Reutens, Julia Hocking

**Affiliations:** ^1^Hotchkiss Brain Institute and Department of Radiology, University of CalgaryCalgary, AB, Canada; ^2^Centre for Advanced Imaging, The University of QueenslandSt. Lucia, QLD, Australia; ^3^Queensland Brain Institute, The University of QueenslandSt. Lucia, QLD, Australia; ^4^Centre for Clinical Research, The University of QueenslandBrisbane, QLD, Australia; ^5^School of Psychology and Counselling, Faculty of Health, Queensland University of TechnologyKelvin Grove, QLD, Australia

**Keywords:** functional MRI, fMRI, BOLD, diffusion MRI, DfMRI, functional imaging, fMRI contrast, visual cortex

## Abstract

Reliance on the hemodynamic response as a surrogate marker of neural activity imposes an intrinsic limit on the spatial specificity of functional MRI. An alternative approach based on diffusion-weighted functional MRI (DfMRI) has been reported as a contrast less reliant on hemodynamic effects, however current evidence suggests that both hemodynamic and unique neural sources contribute to the diffusion signal. Here we compare activation patterns obtained with the standard blood oxygenation level-dependent (BOLD) contrast to DfMRI in order to gain a deeper understanding of how the BOLD proportion contributes to the observable diffusion signal. Both individual and group-level activation patterns obtained with DfMRI and BOLD to a visual field stimulation paradigm were analyzed. At the individual level, the DfMRI contrast showed a strong, positive relationship between the volumes of cortex activated in response to quadrant- and hemi-field visual stimulation. This was not observed in the corresponding BOLD experiment. Overall, the DfMRI response indicated less between-subject variability, with random effects analyses demonstrating higher statistical values at the peak voxel for DfMRI. Furthermore, the spatial extent of the activation was more restricted to the primary visual region for DfMRI than BOLD. However, the diffusion signal was sensitive to the hemodynamic response in a manner dependent on experimental manipulation. It was also limited by its low signal-to-noise ratio (SNR), demonstrating lower sensitivity than BOLD. Together these findings both support DfMRI as a contrast that bears a closer spatial relationship to the underlying neural activity than BOLD, and raise important caveats regarding its utilization. Models explaining the DfMRI signal change need to consider the dynamic vascular contributions that may vary with neural activity.

## Introduction

The accuracy of human brain mapping using functional magnetic resonance imaging (fMRI) is dependent on the spatial coupling between the location of the measured fMRI response and underlying neural activity. Blood oxygenation level-dependent (BOLD) contrast is the dominant technique used to infer neural activity in fMRI, relying on the changes in deoxyhemoglobin concentration and metabolic demands that accompany changes in neural activity (Hoge et al., 1999; Mark et al., 2015). Gradient-echo (GE) echo planar imaging (EPI) is the most widely used method to attain BOLD contrast sensitization, although signal changes may be spatially displaced from the site of neural activity (Turner, 2002; Diekhoff et al., 2011). At magnetic field strengths < 4 Tesla, the signal mainly originates from the surface of the cerebral cortex dominated by large vessels (Kim et al., 2004). BOLD techniques sensitized to the microvasculature aim to improve the proximity of the fMRI signal change to the site of neural activation (Qiu et al., 2012; Huber et al., 2013; Siero et al., 2013), however the fundamental limitation of dependence on hemodynamic changes remains. Because neurovascular-coupling leads to vascular changes that are not confined to the regions with increased neural activity, the hemodynamic response extends beyond regions of cortical activation, reducing spatial specificity for functional localization (Ugurbil et al., 2003).

Activation-associated decrease in water diffusion observed with highly diffusion-sensitized fMRI sequences has been suggested as an alternate technique for brain mapping (Le Bihan et al., 2006). At high *b*-values (>200 s/mm^2^) the intravascular contribution to the signal is thought to be negligible (Le Bihan et al., 1988). Therefore, the advantage of diffusion-weighted fMRI (DfMRI) is that it may reflect a physiological signal that is independent of neurovascular coupling and closer to the source of neuronal activity. Recent experimental studies have provided support for the DfMRI and the BOLD fMRI signal originating mainly from different sources of contrast. It has been shown that the diffusion response is not only temporally distinct, but also precedes the hemodynamic response (Aso et al., 2009, 2013; Williams et al., 2014, 2015). In hippocampal slices devoid of vasculature, cellular changes induced by activation have been detected using a DfMRI sequence (Flint et al., 2009). Decreases in water diffusion accompanying neural stimulation have been detected following the administration of nitroprusside, which is known to eliminate neurovascular coupling (Tsurugizawa et al., 2013). However, other studies have provided evidence for a vascular contribution to DfMRI signal (Miller et al., 2007; Autio et al., 2011; Ding et al., 2012; Kuroiwa et al., 2014). For instance, in one study, modulations to cerebral blood flow independent of changes in neural activity using hypercapnia resulted in detectable DfMRI signal changes (Miller et al., 2007). This finding led authors to conclude that residual vascular contributions to the DfMRI signal dominate the contrast mechanism.

While the exact physiological underpinnings of the DfMRI signal remain uncertain, taken together the current evidence suggests that contributions from both BOLD and non-BOLD sources influence the diffusion signal. However, little attention has been paid to the spatial properties of activation obtained with DfMRI and whether these more closely resemble a contrast dominated by BOLD, or one that is more indicative of a unique signal source. If the latter is correct, then it can be assumed that DfMRI activation patterns would appear more spatially specific to the locus of neural activity. The organization of the primary visual cortex (V1) provides an ideal means to compare DfMRI and BOLD activation patterns. V1 contains a complete retinotopic map of the visual field, where visual stimuli are represented contiguously on the cortex (Wandell et al., 2007). Individually stimulating visual field quadrants modulates activity in anatomically defined regions, superior and inferior to the calcarine sulcus in both cerebral hemispheres (Tootell et al., 1998). Furthermore, physiological responses in V1 to increasingly sized visual stimuli have been described as linear (Hansen et al., 2004). Non-linearity has been found for simultaneously presented stimuli (Pihlaja et al., 2008; Kay et al., 2013), which may be due to the well-characterized feature of surround suppression. This is the reduced response of a neuron to stimuli within the visual field occupying both its central receptive field and immediate surround (Kastner et al., 2001; Nassi et al., 2013). Surround suppression has shown to be more prominent in the extrastriate visual cortices compared to V1, as these neurons have larger receptive fields (Press et al., 2001). Taking together the retinotopic properties of V1 and the findings of spatial linearity, it can be assumed that a positive, linear relationship between spatial extent of visual stimulus and V1 neural response will be found when stimuli are presented to separate visual field quadrants outside the range surround suppression. This positive relationship is expected to be consistent across individuals as it is independent of V1 anatomy, which can show inter-individual size and location variations (Stensaas et al., 1974; Amunts et al., 2000).

The aim of the present study was to compare activation patterns obtained with DfMRI and BOLD. In doing these comparisons, we evaluate the extent to which the BOLD and non-BOLD sources are reflected in the DfMRI signal during neural activity. Activation profiles to left quadrant and left hemi-field visual stimulation were assessed at the group and individual level. At the individual level, we first ran analyses that characterized the location and extent of the activation. The vascular response to neural activity has shown to extend beyond the activated cortical region (Parkes et al., 2005), which may result in BOLD responses to visual stimulation conditions that overlap and/or extend beyond the primary visual regions. Therefore, it was predicted that, if DfMRI activation is largely reliant on physiological sources distinct from BOLD, then the cortical response in the contralateral hemisphere would be more closely related to the area of visual field stimulated for DfMRI than for BOLD. Second, in order to gain a deeper understanding of BOLD and DfMRI activations, the magnitudes of signal changes in different early visual regions were assessed, and magnitudes obtained from lower and upper visual field stimulation were compared. Finally, the temporal characteristics of BOLD and DfMRI signals were compared through the estimation of response functions to visual stimulation. Together these analyses aimed to help develop our understanding of both the BOLD and non-BOLD effects on the diffusion signal in the visual cortex.

## Materials and methods

### Participants

Ten healthy adults aged 19–39 years (mean age = 24.1 years, 5 male) with no history of neurological illness or injury gave written informed consent to participate in this study. All had normal or corrected-to-normal vision. The project was approved by and carried out in accordance with the University of Queensland Medical Research Ethics Committee for human studies.

### Stimulus paradigm

**Figure 1:**
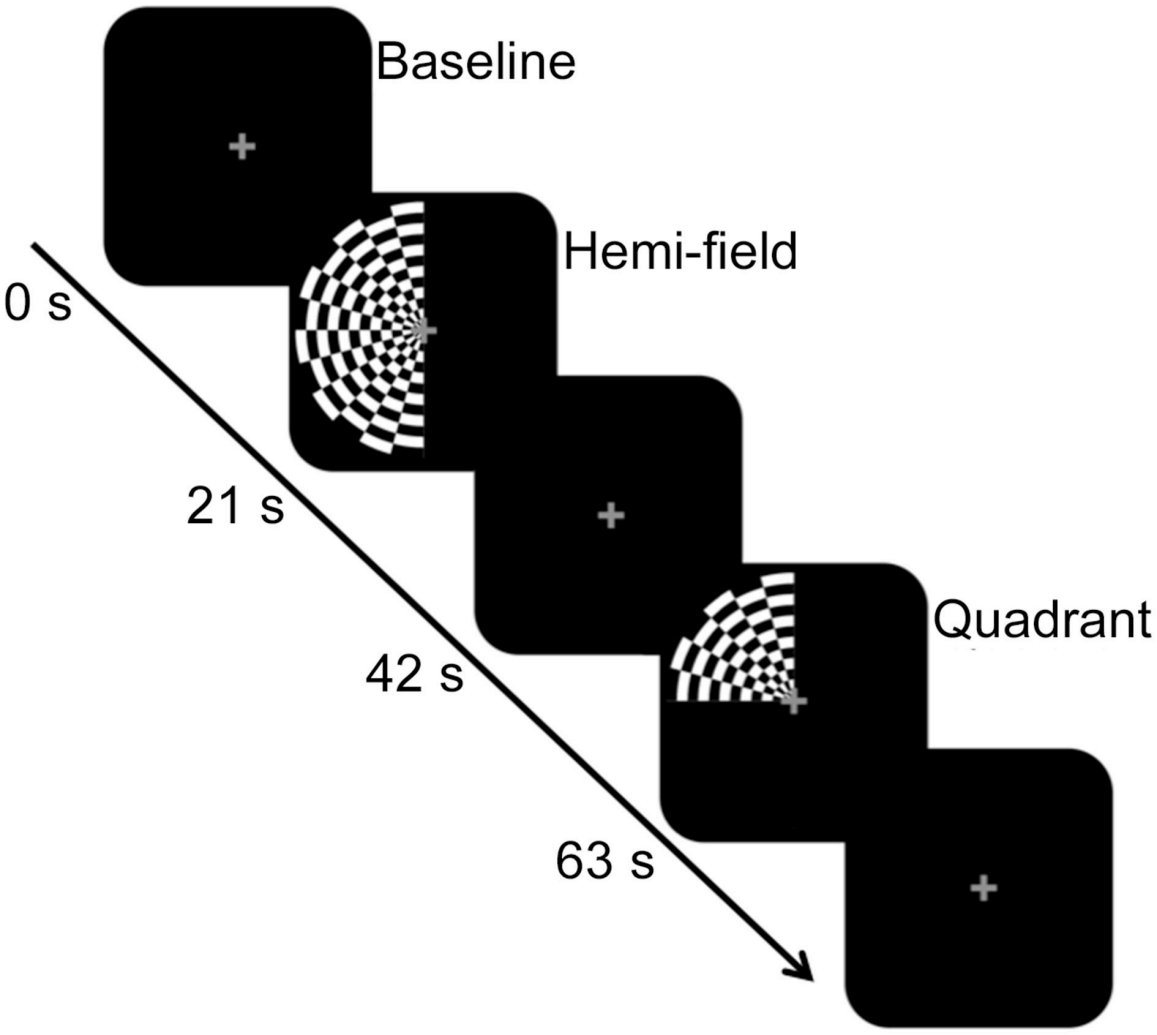
**Schematic diagram of the stimulus paradigm**. The hemi-field and quadrant checkerboard stimuli were presented in a counter-balanced order in blocks of 21 s, and interspersed with a baseline condition. The baseline condition, also presented for 21 s, consisted of a black screen and central fixation cross. The central fixation cross was present across all conditions.

Inside the scanner bore, participants viewed the visual stimulus via a head coil-mounted mirror. The stimulus was back-projected onto an LCD screen located inside the bore of the scanner. The LCD screen was located approximately 83.5 cm from the mirror, which was ~12 cm above the eyes. The stimulus consisted of a full visual field black-and-white radial checkerboard with a contrast reversal rate of 7.5 Hz, and appeared in two experimental conditions. In both conditions, only the left half of the checkerboard was displayed against a black background. For the “hemi” visual field condition (~15.90° visual angle), the entire left half of the checkerboard stimulus was exposed to the left hemi-field, aligned with the vertical meridian. In the “quadrant” visual field condition (~7.95° visual angle), the upper left quadrant of the checkerboard was exposed, aligned with the vertical and horizontal meridian. The eccentricity was equal between the conditions. In both experimental conditions, a gray central fixation cross was consistently present. These experimental conditions were presented in a block design, interspersed with a baseline condition consisting of a black screen and gray central fixation cross. Epoch length for the experimental conditions and baseline condition was 21 s. Figure 1 shows a schematic diagram of the stimulus paradigm and sample stimuli. There were 7 experimental epochs and 7 baseline epochs within each run, with a total of 2 runs for BOLD and 10 runs for DfMRI. For DfMRI, 5 runs contained 3 hemi and 4 quadrant experimental conditions. The other 5 contained 4 hemi and 3 quadrant conditions. For BOLD, there was 1 run of each. The presentation order of the experimental conditions within each run was counter-balanced and pseudo-randomized. The ordering of DfMRI/BOLD runs within the scan session was counterbalanced. There was one single scan session for each participant.

Participants were instructed to focus on the central fixation cross throughout the duration of the experiment. To ensure alertness, participants were instructed to make a button-press with their right hand at the onset and offset of the stimulus, with responses recorded. Participants also gave verbal confirmation of their alertness and ability to fixate on the central cross. The stimulus presentation and behavioral responses was controlled by Cogent and Cogent Graphics (www.vislab.ucl.ac.uk) running on MATLAB (Mathworks, Sherborne, MA, USA).

### Data acquisition

All images were acquired on a Siemens 3 T TIM Trio (Siemens Medical Solutions, Erlangen, Germany) with a 12-channel birdcage head coil. Extra padding was inserted to minimize head movement, and participants were instructed to remain stationary. The DfMRI acquisition was a twice-refocused spin-echo echo-planar image (EPI) sequence, with diffusion sensitization attained by the addition of an interleaved pair of bipolar magnetic field gradients with a *b*-value of 1800 mm/s^2^ (Le Bihan et al., 2006). BOLD sensitized images were acquired with a T_2_^*^-weighted EPI sequence. The TR was 1500 ms for both functional sequence types, and the TE was 92 and 36 ms for DfMRI and BOLD, respectively. The voxel size was 3 × 3 × 3 mm with 10 slices separated by a 50% gap acquired in an interleaved order. In each functional run, 200 partial brain volumes centered on the calcarine sulcus and covering the entire occipital cortex were acquired. Five DfMRI volumes from every run were set to *b* = 0 and were therefore excluded from all analyses, resulting in 195 DfMRI volumes collected for each run. These images were collected prior to stimulus onset, and were only included to improve registration between functional and anatomical runs, if required. A high-resolution T_1_-weighted structural image was also collected for each scan session (TR = 1900 ms, TE = 2.32 ms, FOV = 230 × 230, 0.9 mm^3^ isotropic voxels). The total scan time for every participant in each experimental session was 64 min.

### Data analysis

Statistical Parametric Mapping 8 (SPM8) (Wellcome Trust Centre for Neuroimaging, London UK) running on MATLAB was used to analyze the images. DfMRI and BOLD images were preprocessed separately. Preprocessing followed standardized procedures. Images were slice time corrected, and realigned and resliced using a 6-parameter rigid body spatial transformation (Friston et al., 1995). The structural scan was coregistered to the mean functional image for each participant, and normalized to the MNI template using the Unified Segmentation algorithm (Ashburner and Friston, 2005). Accuracy of coregistration between DfMRI and anatomical images was assessed by careful visual inspection for all participants. Spatial smoothing using a 6 mm FWHM Gaussian kernel was performed on the normalized images. Both the smoothed and unsmoothed normalized images were analyzed.

### Number of runs

In each scan session, 10 × 5 min runs of DfMRI and 2 × 5 min runs of BOLD were collected and entered into the first-level analyses. This ratio was essential given the low signal-to-noise ratio (SNR) of DfMRI. Previously, it had been shown that temporal SNR (tSNR) is one of the essential factors in predicting whether a study will be successful in detecting activation (Murphy et al., 2007). The authors of this paper provided a formula that calculates the estimated number of time points required for signal detection within a single voxel, at a given *P*-value for the tSNR and expected effect size. This formula was implemented here with calculated tSNR, a linear range of *P*-values and effect sizes for both DfMRI and BOLD as input, in order to highlight the disparity between DfMRI and BOLD in terms signal detection power. Furthermore, we aimed to highlight the need for more DfMRI data relative to BOLD. The formula provided by Murphy et al. (2007):

$$\begin{array}{l} N_{TP}\;=8{\left[1.5\;\left(1+e^{{\text{log}}_{10}\text{P}∕2}\right)\left(\frac{{\text{erfc}}^{-1}\;\left(\text{P}\right)}{\left(\text{tSNR}\right)\left(\text{eff}\right)}\right)\right]}^{2} \end{array}$$

Where number of time points (*N*_*TP*_) for detection of activation can be calculated when the tSNR and anticipated effect size (eff) are known. The inverse complementary error function and *P*-values are given here by (erfc^−1^) and (P) respectively. Here, tSNR was calculated from the right primary visual cortex for both DfMRI and BOLD. We calculated tSNR maps from one run of functional data that had undergone correction for motion and slice timing. To create tSNR maps, the mean signal of the fMRI time series was divided by the standard deviation (Welvaert and Rosseel, 2013) using tools in FSL (Smith et al., 2004). The mean tSNR within our primary anatomical region-of-interest (ROI), right V1, was calculated from each subject's tSNR map for BOLD and DfMRI. The mean tSNR was 51.3 (±10.9) for BOLD and 9.3 (±0.6) for DfMRI. For the present calculations, these mean tSNR values were entered into the equation.

Based on prior work (Aso et al., 2009; Williams et al., 2014), the expected effect size was 1% for DfMRI when using the diffusion-hemodynamic response function (DhRF) described by Aso et al. (2009). For the purpose of the current calculations, the anticipated BOLD effect size was also input as 1%. However, this is a conservative estimate and BOLD effect size could be expected to be as large as 5%, considering previous work (Williams et al., 2015).

**Figure 2:**
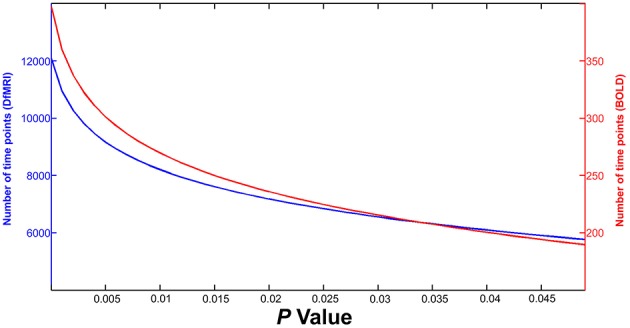
**Results of calculations estimating number of time points required for guaranteed detection of activation within a single voxel**. DfMRI (blue line, Y-axis left) and BOLD (red line, Y-axis right) time points are shown for increasing *P*-value (X-axis).

This equation was entered into MATLAB and calculations were performed to estimate the *N*_*TP*_ required for signal detection for both DfMRI and BOLD with increasing *P*-value, given the expected tSNR and effect sizes. As shown in Figure 2, at the most liberal *P*-value, approximately 200 time points would be required for BOLD activation detection within a single voxel (red line and right Y-axis). At the same *P*-value, ~30 times as much DfMRI data would need to be collected in order to have adequate power to detect diffusion activation (blue line and left Y-axis). Considering the overly conservative effect size implemented here for BOLD, it can be assumed that this is an overestimation of anticipated BOLD time points. However, the same cannot be said for DfMRI, which would require the collection of an impractically large number of time points considering its low tSNR. These calculations exemplify the need for more DfMRI time points, given its low tSNR. According to the above equation however, a larger effect size for BOLD would mean that even if tSNR were comparable between the two contrasts, the BOLD acquisition would still require fewer time points for signal detection than DfMRI. Different statistical thresholds for DfMRI and BOLD may be necessary when a large difference in effect size is anticipated. In the following analyses, we implemented identical statistical thresholds for DfMRI and BOLD when possible. However, when a reduction in effect size is anticipated, a more liberal threshold for DfMRI may be required.

### First-level analyses

**Figure 3:**
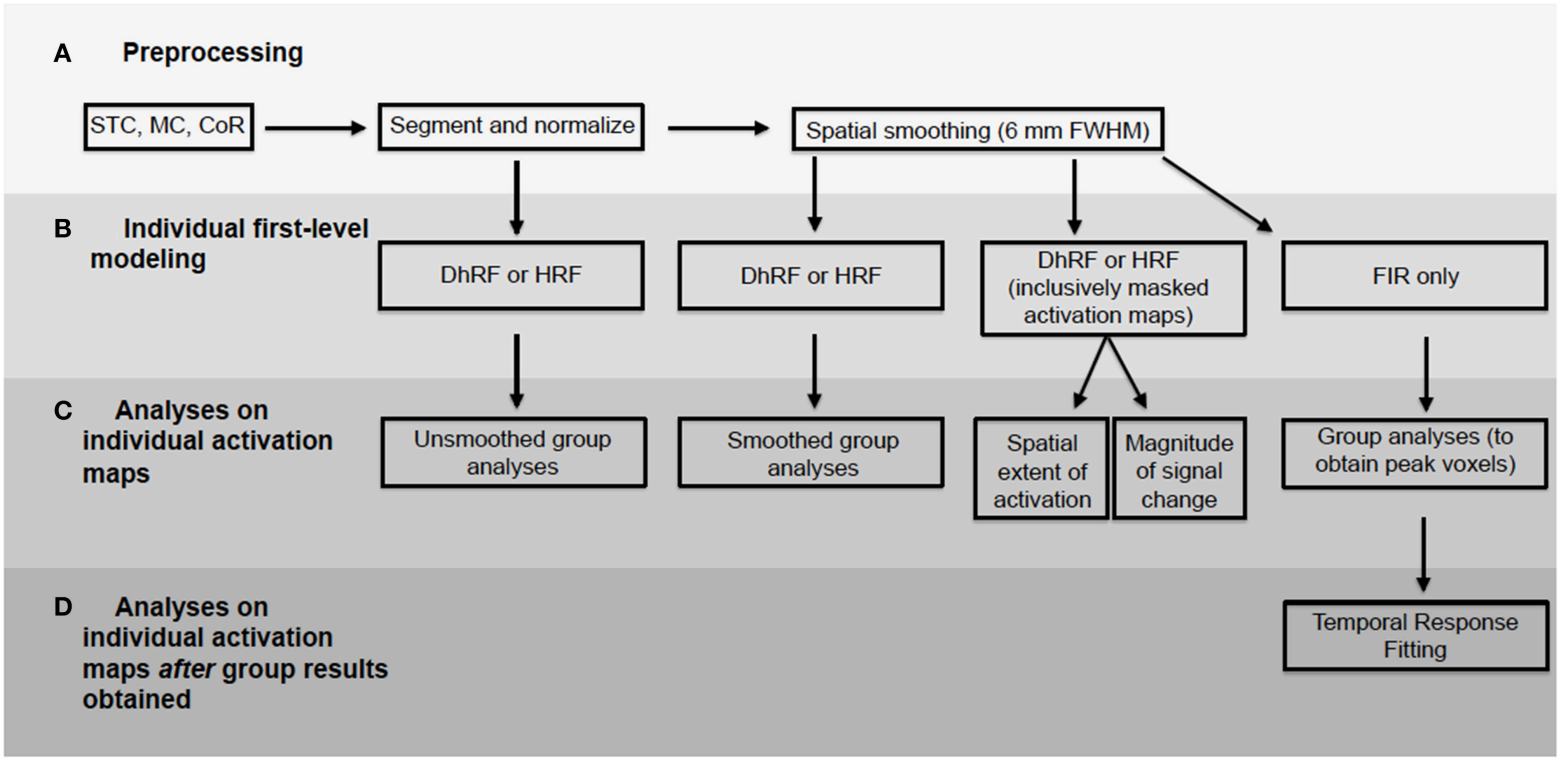
**Flowchart demonstrating all data analyses performed**. BOLD and diffusion images analyzed separately. Processing pipeline included **(A)** preprocessing, **(B)** first-level modeling, **(C)** analyses performed on activation maps from first-level and **(D)** analyses performed on first-level maps using peak voxels obtained from group-level results. STC, slice timing correction; MC, motion correction; CoR, coregistration.

For first-level analyses, DfMRI and BOLD were analyzed separately. The 2 BOLD runs obtained from each participant were modeled together. This was achieved using the canonical HRF convolved with a boxcar function equal to the length of the checkerboard stimulation. For the DfMRI data, the 10 runs obtained from each participant were modeled together using the DhRF. This too was convolved with a boxcar function equal to the length of the checkerboard stimulus. For each of the four experimental conditions corresponding to quadrant visual field stimulation for DfMRI (DfMRI_quadrant_) and BOLD (BOLD_quadrant_) and hemi-field stimulation for DfMRI (DfMRI_hemi_) and BOLD (BOLD_hemi_), first-level contrast images relative to baseline were computed. These first-level analyses were performed twice for each participant, once with the normalized images that had been spatially smoothed, and again for the normalized images without smoothing. A diagram showing all data analyses performed here is demonstrated in Figure 3.

### Group analyses

Spatial smoothing was applied to increase the SNR of DfMRI and allow for comparisons of DfMRI and BOLD activation patterns. For consistency, the same size smoothing kernel was applied to both DfMRI and BOLD. However, we also wanted to precisely compare the spatial localization of the sequences across participants without the use of smoothing. Therefore, random-effects group analyses were performed separately for both the unsmoothed and smoothed normalized images, for DfMRI and BOLD. These analyses involved entering first-level contrast images from the individual analyses into one-way ANOVAs.

All group activation maps obtained from the unsmoothed images were thresholded at *P* < 0.05 false discovery rate (FDR) corrected at the voxel-level. Group activation maps calculated from the 6 mm FWHM smoothed images were thresholded at *P* < 0.05 family wise error (FWE) corrected for multiple comparisons (Worsley et al., 1996).

### Analyses of individual activation patterns

The first-level activation maps from the smoothed images were used in further analyses comparing DfMRI and BOLD activation patterns. For all analyses described below, unless otherwise stated, the statistical maps were thresholded at *P* < 0.05 FWE. Identical statistical thresholds were applied to DfMRI and BOLD activation maps in all analyses except for the temporal response fitting. Statistical tests outside of SPM8 and MATLAB were performed using IBM SPSS Statistics 20 (IBM Corp., Armonk, USA).

#### Spatial extent of activation

The spatial extent of the activation pattern elicited within V1 by each of the four experimental conditions (DfMRI_hemi_, DfMRI_quadrant_, BOLD_hemi_, BOLD_quadrant_) was defined as the number of voxels activated within the right primary visual cortex for each stimulation condition. As visual field stimulation was unilateral, only contralateral V1 was considered in the analysis. The first-level SPM images contrasting each condition relative to baseline were used in this analysis. The SPM8 inclusive masking procedure was utilized to restrict results to the right V1, defined from an anatomical ROI obtained from the SPM Anatomical Toolbox (Amunts et al., 2000; Eickhoff et al., 2005, 2006). This method defines an anatomical ROI from a maximum probability map (MPM), which is a single representation of multiple probabilistic cytoarchitectonic maps. Each voxel in the MPM is assigned to a cytoarchitectonic area based on its spatial position. The anatomical ROI implemented here was defined through the MPM using a binary method. All voxels assigned to Area 17 (primary visual cortex) in the MPM were set to a value of 1 and all outside voxels set to 0. Creating anatomical ROIs using this method has been demonstrated as highly accurate in regards to anatomical specificity, compared to thresholded probabilistic maps (Eickhoff et al., 2006). Anatomical ROIs were output in MNI template space, necessitating the spatial normalization of each subject's brain to MNI space. There were 671 fMRI voxels within the anatomical mask. The numbers of activated voxels within the right V1 for each of the four experimental conditions were extracted from the inclusively masked images. While voxels outside of V1 and thus outside the mask were expected to activate, we chose to limit the analysis to voxels within V1 as this region contains a retinotopically organized map of the entire visual field (Engel et al., 1997). To assess the relationship between increasing area of visual field stimulation and spatial extent of the activation, these voxel numbers were then entered into bivariate regression analyses. Within-sequence (DfMRI_hemi_−DfMRI_quadrant_; BOLD_hemi_−BOLD_quadrant_) analyses were performed, in addition to within-visual stimulation (DfMRI_hemi_−BOLD_hemi_; DfMRI_quadrant_−BOLD_quadrant_) comparisons.

#### Magnitude of signal change

To compare DfMRI and BOLD response amplitudes to visual stimulation, the percent signal change was calculated and compared between all experimental conditions in areas V1 and V2 separately. Area V2 was included in this analysis to allow for a more thorough interrogation of the DfMRI signal and comparison to BOLD across multiple visual regions. Percent signal changes to quadrant and hemi-field conditions were calculated from ROIs defined from BOLD and DfMRI activation maps, and atlas-defined anatomical masks, on a per-subject basis. For this analysis, it was important to include as many voxels as possible in the calculation of percent signal change to obtain accurate measurements. However, it was also important to avoid voxels with contributions from larger vasculature. These would be expected to artificially inflate the BOLD but not the DfMRI magnitude. To achieve this, individual activation maps isolating the effects of visual stimulus (hemi or quadrant) relative to baseline were generated to include only voxels within the right V1 or V2. To restrict voxels to either V1 or V2, the inclusive masking procedure described above was implemented. The same V1 anatomical mask described in the previous analysis was used to calculate percent signal change in area V1. An anatomical V2 mask was created from the same MPM as V1, also using the SPM Anatomical Toolbox (Amunts et al., 2000; Eickhoff et al., 2005, 2006). ROIs from which percent signal change were calculated were the intersecting voxels activated in both DfMRI and BOLD inclusively masked activation maps. This ensured that only voxels commonly activated in both BOLD and DfMRI conditions were included in the analysis. Maps were thresholded at *P* < 0.05 FDR corrected for both DfMRI and BOLD. This was reduced from the more conservative FWE-corrected threshold in order to increase the number of commonly activated voxels.

For each individual, the percent signal change in V1 and V2 was calculated using the MarsBaR toolbox for SPM (Brett et al., 2002). Two separate series of analyses were then performed in IBM SPSS on the calculated percent signal changes. First, bivariate correlation analyses were performed within (DfMRI_hemi_− DfMRI_quadrant_; BOLD_hemi_− BOLD_quadrant_) and between (DfMRI_hemi_− BOLD_hemi_; DfMRI_quadrant_− BOLD_quadrant_) sequence types for both V1 and V2 regions, in order to establish whether inter-individual variation correlated across conditions. Second, to determine if there was any age effect on BOLD and DfMRI signals, the relationship between participant age and percent signal change was assessed. Because the age distribution of the study participants was highly skewed, a series of Spearman's rank-order correlations were conducted.

#### Lower vs. upper visual field stimulation

Previous research has demonstrated visual field asymmetries with regards to lower vs. upper visual field processing, with stronger neural responses arising from cells with receptive fields in the lower visual field (Hagler, 2014). Further analyses on signal magnitude were performed to determine whether DfMRI or BOLD signal changes reflect this asymmetry. In order to isolate voxels responding preferentially to the lower visual field, first-level activation maps with *t* contrasts identifying voxels responding significantly more to hemi-field relative to quadrant stimulation were created. All maps were thresholded at *P* < 0.05 FDR-corrected. Identical to the previous signal magnitude analysis, only voxels that were activated for both DfMRI and BOLD were used as ROIs. The percent signal change to the hemi-field visual stimulus was then calculated from these ROIs on a per-subject basis, in order to obtain measurements of signal magnitude to lower visual field stimulation. These lower field signal magnitudes were compared to upper visual field signal magnitudes, which were obtained from the quadrant conditions (both areas V1 and V2) described above. The ROIs corresponding to the lower field were not split into V1 and V2, due to low voxel count.

A series of paired samples *t*-tests were conducted to determine if the voxels preferentially responding to the lower visual field produced larger signal changes than the voxels responding to the upper visual field. These *t*-tests were performed within sequence (DfMRI_lower_− DfMRI_upper_; BOLD_lower_− BOLD_upper_) only. Both visual regions (V1 and V2) for the upper visual field condition were included in the analysis.

#### Temporal response fitting

To characterize the temporal response profiles to each of the visual stimulation conditions, subject-specific impulse response functions were estimated for each participant. The voxel selection process for time-course extraction aimed to identify voxels responding preferentially to each stimulus while avoiding selection bias. To select voxels for the fitting analysis in an unbiased manner, first-level analyses were performed specifically for voxel selection implementing the finite impulse response (FIR) basis functions (see Figure 3). This allowed variability in the shape and the timing parameters of the impulse response without imposing an *a priori* functional form, unlike the prior first-level analyses using the canonical HRF/DhRF to model the BOLD and DfMRI response respectively. These first-level analyses using the FIR functions resulted in contrast images calculated from the linear combination of beta images. Second-level group analyses were then performed separately for DfMRI and BOLD. This allowed for the peak activation to each visual stimulation condition relative to baseline at the group level to be identified. Time courses were then extracted from the corresponding individual statistical maps using 10 mm spherical volume of interests (VOIs) centered on the group peak maxima. The time courses of the significant voxels within this VOI were extracted for every run of data for every participant. For BOLD, the individual maps were thresholded at *P* < 0.05 FWE corrected for multiple comparisons. Because the FIR was used, the diffusion maps were set to an uncorrected threshold of *P* < 0.001. The lowered threshold was selected because the use of this flexible model was expected to reduce activation detection in the more noisy DfMRI data.

Estimating the subject-specific hemodynamic response functions was achieved using code from the sHRF Toolkit running on MATLAB (Shan et al., 2014). This code is openly available for download at http://www.cai.uq.edu.au/shrf-toolkit. The BOLD and DfMRI response functions were modeled as the sum of two gamma functions, similar to previous work estimating the DhRF and HRF (Handwerker et al., 2004; Aso et al., 2013; Beers et al., 2015). These were convolved with the stimulus paradigm modeled as a series of boxcar functions to create a simulated time-course. Fitting between the simulated time-course and raw data was performed using non-linear optimization to find the parameters of the modeled impulse response function that minimized the residual sums of squares (RSS) between the simulated and real data. For both DfMRI and BOLD, fitting was initialized using the parameters of the canonical HRF with six free parameters (Shan et al., 2014). This was performed for every run of data for each participant. Runs of data with parameters exceeding one standard deviation from the group mean were excluded as outliers. Surviving parameter estimates were averaged across runs for each participant to provide a robust subject-specific response function. The time-to-peak (TTP), width of the positive response, onset delay and the area under the curve (AUC) were calculated for each subject-specific response function. The definitions of these features are outlined in Shan et al. (2014). These values were then entered into a repeated-measures two-way analysis of variance (ANOVA), with factors of parameter (TTP, width, onset delay, AUC) and experimental condition (DfMRI_quadrant_, DfMRI_hemi_, BOLD_quadrant_, BOLD_hemi_). *Post-hoc* analyses were performed using repeated-measures one-way ANOVAs and paired comparisons using Fishers's least significant difference. To compare the goodness-of-fit of the predicted and measured response across conditions, RSS error values were compared between the four conditions using a non-parametric repeated-measures Friedman's ANOVA.

## Results

### Individual analyses

For DfMRI, spatial smoothing improved signal detection. The unsmoothed first-level activation maps were more scattered, showing much smaller but a greater number of clusters for DfMRI at *P* < 0.05 (FDR corrected) relative to the smoothed images at an *P* < 0.05 FWE corrected threshold. The difference between the smoothed and unsmoothed BOLD individual activation maps was less pronounced. For the smoothed images, first-level activation maps resulted in robust activation within the right occipital lobe for all four experimental conditions (DfMRI_quadrant_, DfMRI_hemi_, BOLD_quadrant_, BOLD_hemi_) relative to baseline. BOLD demonstrated larger cluster sizes than DfMRI. This was consistent across all participants. At the FWE corrected threshold, the smoothed DfMRI data showed smaller cluster sizes than BOLD however activation was consistently localized to the upper and lower banks of the calcarine sulcus for the hemi field condition, and to the lower bank for the quadrant field condition. One participant showed relatively small peak cluster sizes for BOLD, as shown in Table 1B. Peak location in MNI coordinates, its corresponding *t*-value and the peak cluster size are shown in Table 1A for DfMRI and Table 1B for BOLD for each first-level analysis using the smoothed and unsmoothed images.

**Table 1:** **(A) Whole-volume brain activation observed for the DfMRI conditions relative to baseline; (B) Whole-volume brain activation observed for the BOLD conditions relative to baseline**.

	**DfMRI_hemi_**										**DfMRI_quadrant_**									
***Pt***	**FWHM (6 mm)**					**Unsmoothed**					**FWHM (6 mm)**					**Unsmoothed**				
	**Peak**			***t***	***k***	**Peak**			***t***	***k***	**Peak**			***t***	***k***	**Peak**			***t***	***k***
**A**																				
1	12	−94	−2	9.5	171	12	−94	−2	6.2	55	9	−88	−14	7.5	115	6	−88	−14	6.2	8
2	15	−94	4	9.0	79	15	−91	7	6.4	40	18	−91	7	5.6	20	12	−82	1	4.5	2
3	9	−97	−11	7.2	101	3	−91	−5	5.4	18	6	−97	−14	6.2	39	27	−91	−14	4.4	1
4	9	−85	−8	8.3	157	6	−88	−8	6.0	80	9	−85	−5	7.6	53	6	−85	−5	5.1	19
5	15	−97	7	9.2	354	15	−97	4	7.0	259	9	−91	4	7.3	127	9	−91	4	6.7	41
6	9	−88	1	9.9	334	12	−79	2	7.2	150	9	−88	−2	9.7	113	9	−88	−11	6.6	30
7	9	−85	−2	6.7	119	9	−85	−2	5.1	11	9	−85	−2	7.6	59	9	−85	−5^*^	4.0	3
8	12	−88	1	10.1	418	15	−79	−14	8.0	291	12	−85	−2	9.5	162	15	−17	−15	7.0	59
9	15	−88	−11	5.8	45	21	−79	−14	4.4	1	24	−79	−14	4.8	4	12	−88	−11^*^	4.5	4
	**BOLD_hemi_**										**BOLD_quadrant_**									
***Pt***	**FWHM (6 mm)**					**Unsmoothed**					**FWHM (6 mm)**					**Unsmoothed**				
	**Peak**			***t***	***k***	**Peak**			***t***	***k***	**Peak**			***t***	***k***	**Peak**			***t***	***k***
**B**																				
1	12	−94	1	29.7	3179	12	−94	19	31.0	1292	9	−82	−17	27.4	674	15	−85	−17	25.4	430
2	15	−91	4	33.3	2944	12	−88	4	33.5	1689	6	−79	−8	22.5	1483	6	−85	−5	23.5	722
3	9	−85	−8	22.2	1328	6	−85	−8	21.8	1185	9	−85	−8	30.9	1208	6	−85	−8	30.3	1187
4	24	−82	−14	24.0	1554	6	−82	−5	24.8	2241	6	−76	−5	25.1	921	6	−82	−5	24.4	955
5	18	−91	10	25.6	2173	12	−91	16	26.2	2354	18	−91	−5	31.9	3103	18	−91	−5	34.7	3257
6	9	−88	−2	36.0	1914	6	−91	1	33.0	2020	9	−88	−5	33.8	1112	12	−88	−5	32.3	1235
7	18	−94	4	39.4	2675	18	−94	4	40.3	3417	6	−85	1	24.9	765	6	−85	1	29.3	1085
8	18	−91	4	33.6	3916	18	−91	1	35.4	4393	12	−82	−5	28.6	2579	12	−85	−8	31.2	2813
9	15	−85	−8	5.4	19	12	−85	−8	6.1	58	−6	−28	79	5.6	36	21	−88	−11	5.5	98

*FWHM (6 mm), smoothed images; Unsmoothed, unsmoothed images; Pt, participant; Peak, MNI coordinates of peak voxel; t, t-value of peak voxel; k, size of peak cluster. SPMs thresholded at P < 0.05 FWE-corrected*.

* *Indicates SPM threshold had to be reduced to P < 0.001 uncorrected for detection of suprathreshold voxels*.

### Group analyses

#### Unsmoothed images

**Figure 4:**
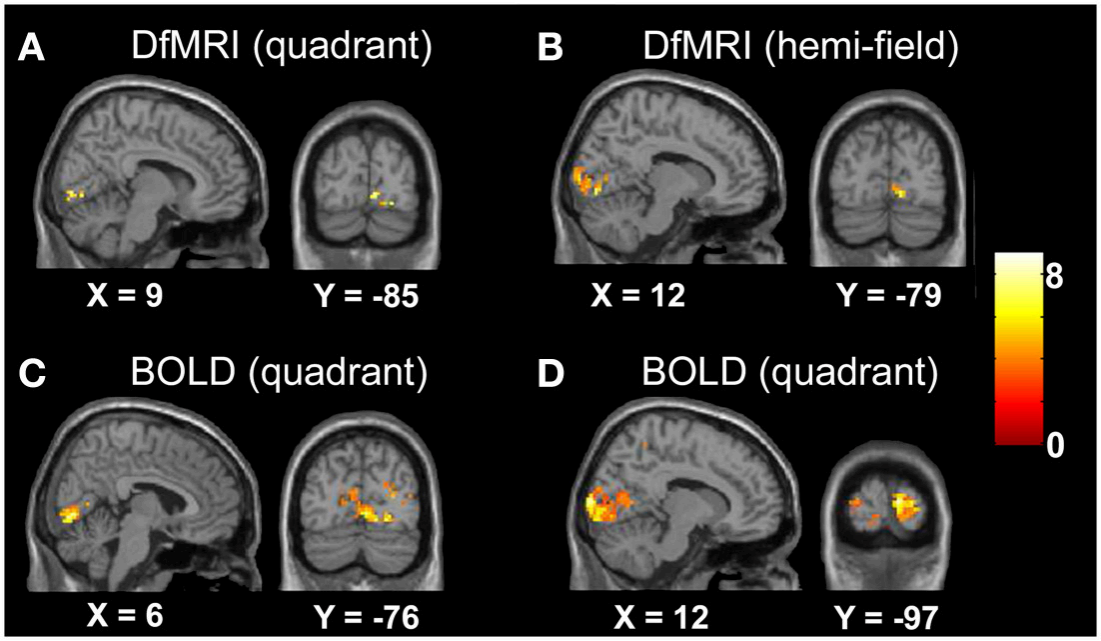
**Results of the group random-effects analyses performed on the unsmoothed images**. Activation maps show visual stimulation relative to fixation contrast for **(A)** DfMRI_quadrant_, **(B)** DfMRI_hemi_, **(C)** BOLD_quadrant_, and **(D)** BOLD_hemi_. All slices shown at level of peak voxel for each condition. Coordinates in standard MNI space. All SPMs overlaid onto the MNI single-subject T_1_-weighted template image and at *P* < 0.05 FDR corrected. Color bar indicates *T*-value.

The random-effects analyses using the unsmoothed images, thresholded at *P* < 0.05 FDR corrected, demonstrated smaller cluster sizes for DfMRI than BOLD. Both sequences showed good spatial localization to the lower banks of the calcarine gyrus for the quadrant condition, and to the upper and lower banks of the calcarine gyrus for the hemifield condition. The BOLD conditions showed some ipsilateral activation. This was not observed in the DfMRI conditions, as shown in Figure 4. This figure is shown in the sagittal and coronal planes at the level of the peak voxel for DfMRI_quadrant_ ([9, −85, −2], *t* = 7.66), DfMRI_hemi_ ([12, −79, −5], *t* = 7.87), BOLD_quadrant_ ([6, −76, −2], *t* = 8.06), and BOLD_hemi_ ([12, −97, 7], *t* = 8.59).

#### Smoothed images

**Figure 5:**
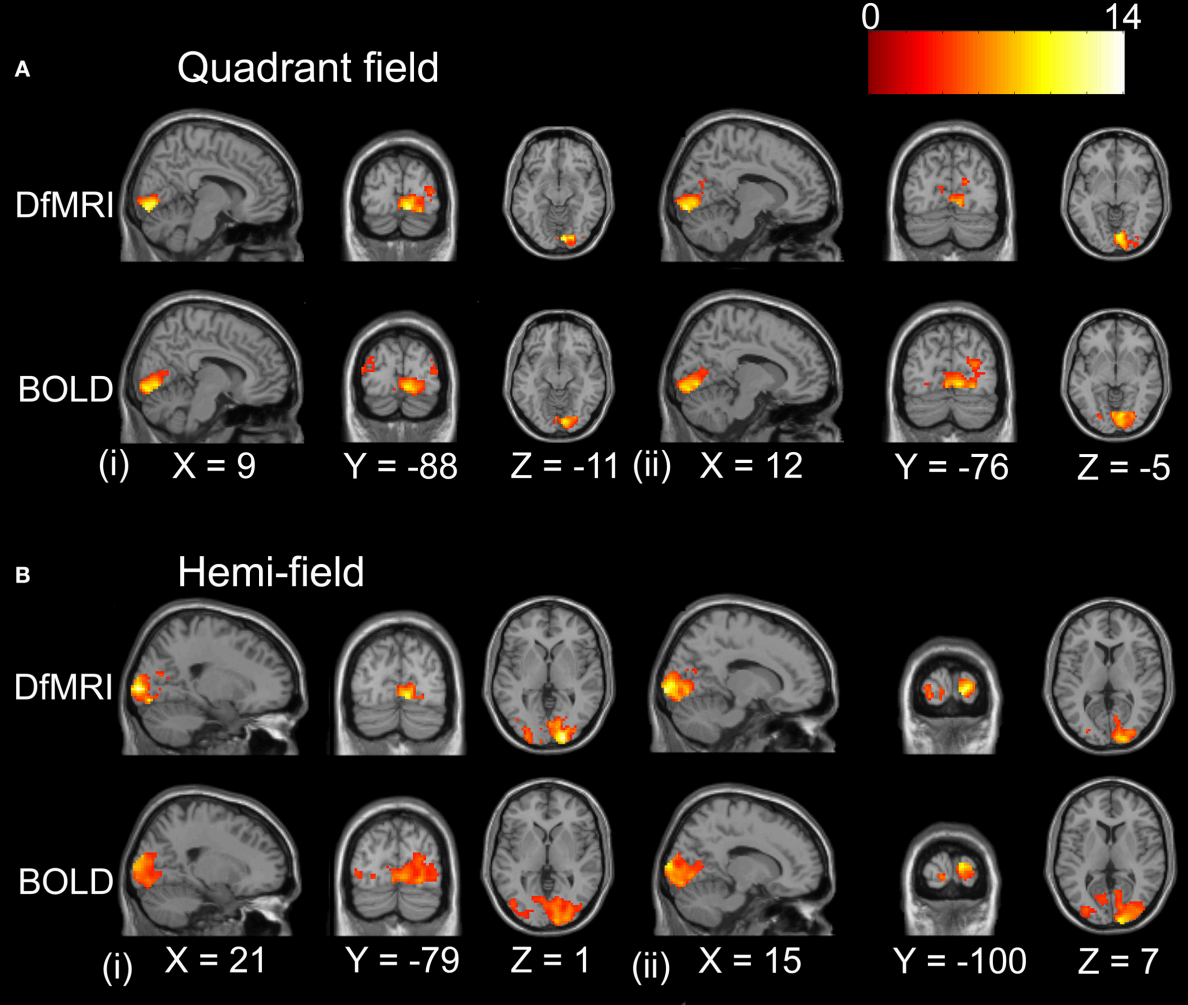
**Results of the group random-effects analyses performed on the smoothed images**. Activation maps show visual stimulation relative to fixation contrast. **(A)** Top panel displays DfMRI_quadrant_ and BOLD_quadrant_ with slices shown at level of (i) DfMRI quadrant peak voxel [9, −88, −11] and (ii) BOLD quadrant peak voxel [12, −76, −5]. **(B)** Bottom panel displays DfMRI_hemi_ and BOLD_hemi_ with slices shown at level of (i) DfMRI hemi peak voxel [21, −97, 1] and (ii) BOLD hemi peak voxel [15, −100, 7]. Coordinates in standard MNI space. All SPMs overlaid onto the MNI single-subject T_1_-weighted template image and at *P* < 0.05 FWE corrected. Color bar indicates *T*-value.

For the random-effects analyses performed on the smoothed images, the highest observed peak *t*-value was obtained for the DfMRI_hemi_ condition ([21, −97, 1], *t* = 13.85), followed by DfMRI_quadrant_ ([9, −88, −11], *t* = 11.68). As demonstrated in Figure 5, DfMRI shows highly significant voxels both at the DfMRI peak voxels, and at the BOLD_hemi_ ([15, −100, 7], *t* = 9.59) and BOLD_quadrant_ ([12, −76, −5], *t* = 9.8) peaks. Moreover, this figure demonstrates the comparable cluster sizes between DfMRI and BOLD in this random-effects analysis. It also shows that for the group analyses performed on the smoothed images, minor ipsilateral activation was observed for both DfMRI and BOLD. All *P* < 0.05 FWE corrected.

### Analyses of individual activation patterns

#### Spatial extent of activation

**Figure 6:**
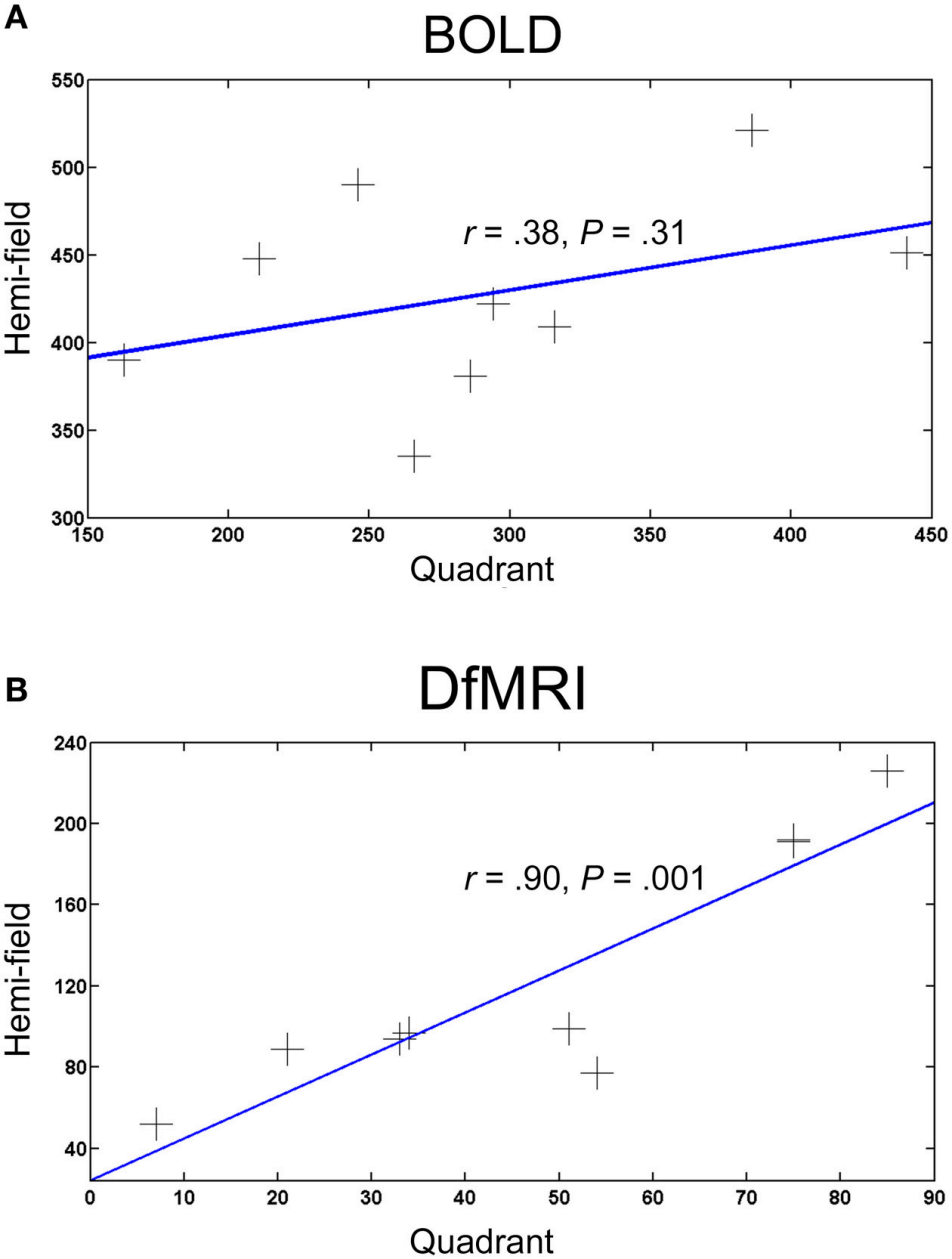
**Scatter plots showing the relationship between the number of activated voxels and visual field stimulation for (A) BOLD and (B) DfMRI**. Each point represents one participant (*N* = 9), and the X and Y-axes represent the number of activated voxels for the quadrant and hemi visual field stimulation conditions, respectively.

To compare the spatial extent of the activation patterns for each condition, the number of voxels activated (smoothed images at a *P* < 0.05 FWE corrected threshold) by the hemi and quadrant visual field stimulation conditions were obtained. One participant failed to show activation within the inclusive masks at the corrected threshold for all four conditions and was excluded from the analysis. Correlation coefficients revealed a significant positive relationship between the DfMRI_quadrant_ and DfMRI_hemi_ conditions, *r* = 0.90, *P* = 0.001. There was no significant relationship between BOLD_quadrant_ and BOLD_hemi_, *r* = 0.38, *P* = 0.31. The two hemi conditions (*r* = 0.46, *P* = 0.22) and the two quadrant conditions (*r* = 0.58, *P* = 0.11) also failed to reach significance. The bivariate regression analysis for DfMRI showed that the number of voxels in the DfMRI_quadrant_ condition significantly predicted the number of voxels in the DfMRI_hemi_ condition [*b* = 2.1, *t*_(8)_ = 5.32, *P* = 0.001]. A significant proportion of the variance in the DfMRI_hemi_ condition was explained by the DfMRI_quadrant_ condition (*R*^2^ = 0.802). The BOLD bivariate regression analysis showed that the number of voxels in the BOLD_quadrant_ condition did not significantly predict the number of voxels in the BOLD_hemi_ condition [*b* = 0.257, *t*_(8)_ = 1.09, *P* = 0.31]. The proportion of variance in the BOLD_hemi_ condition explained by the BOLD_quadrant_ condition was 14.6%. Scatter plots in Figure 6 summarize the relationship between visual stimulation conditions for DfMRI and BOLD separately.

#### Magnitude of signal change

**Figure 7:**
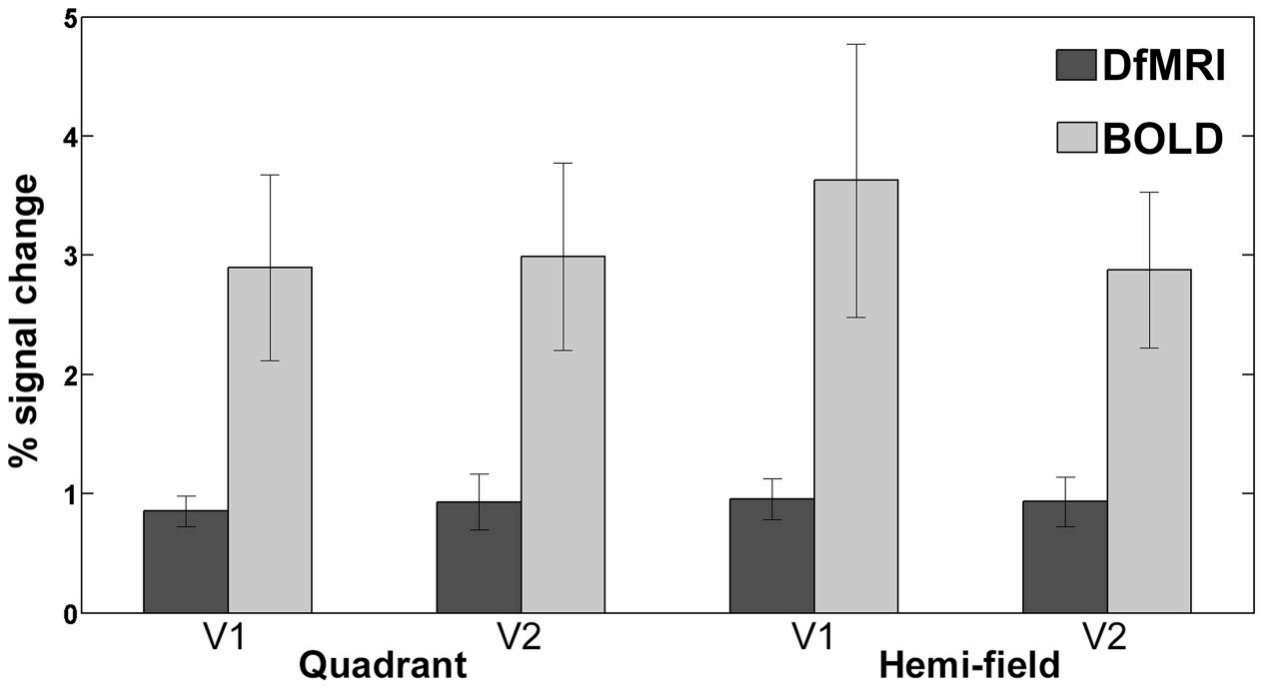
**Bars representing mean percent signal change to both visual stimulation conditions in areas V1 and V2**. DfMRI shown in darker shade, and BOLD in lighter shade of gray. Error bars demonstrate ±1 standard deviation.

The magnitude of the signal change to visual stimulation was compared across all four conditions for both areas V1 and V2. One participant was excluded from this analysis as an outlier, as the calculated percent signal change was greater than two standard deviations from the group mean for the BOLD conditions. As shown in Figure 7, the mean and range of percentages was larger for BOLD for both visual regions. The greatest signal change was observed in V1 for the BOLD_hemi_ condition (3.6 ± 1.1%). The corresponding signal change for this condition in area V2 was slightly lower (2.9 ± 0.66%). The BOLD_quadrant_ conditions saw similar signal change across V1 (2.9 ± 0.78%) and V2 (3.0 ± 0.79%). For DfMRI, a similar pattern was seen where the mean percent signal change for the hemi condition in V1 (0.95 ± 0.17%) was the highest overall, relative to the same condition in area V2 (0.93 ± 0.20%), the V1 activation to the quadrant-field condition (0.85 ± 0.13%), and DfMRI_quadrant_ in V2 (0.93 ± 0.23%).

The bivariate correlation analyses showed a significant relationship between the DfMRI conditions in area V1 (DfMRI_quadrant_ and DfMRI_hemi_, *r* = 0.83, *P* = 0.006) and V2 (DfMRI_quadrant_ and DfMRI_hemi_, *r* = 0.82, *P* = 0.007). No other comparison reached significance (at the 0.01 level, two-tailed). The comparisons between DfMRI and BOLD did not reach significance. The Spearman rank-order correlations showed a significant negative relationship with age and BOLD percent signal change for the hemi-field condition in V1 (*r*_s_ = −0.82, *P* = 0.007). The BOLD percent signal change for this condition decreased with increasing age. The corresponding BOLD condition in area V2 showed a trend toward a relationship with age, but did not reach significance at the two-tailed 0.01 level (*r*_s_ = −0.72, *P* = 0.03). No other condition demonstrated a significant relationship with age.

#### Lower vs. upper visual field stimulation

For voxels preferentially responding to stimulation to the lower visual field, the BOLD mean percent signal change was 3.1 (± 1.1%). The corresponding DfMRI mean was 0.93 (± 0.32%). These means were slightly higher than those described in the previous analysis: the percent signal change for BOLD_quadrant_ and DfMRI_quadrant_ conditions in V1 (2.9 ± 0.78 and 0.85 ± 0.13%, respectively) and V2 (3.0 ± 0.79 and 0.93 ± 0.23%, respectively). Despite this, the difference in percent signal change for voxels preferentially responding to the lower vs. the upper visual field failed to reach statistical significance for all paired comparisons.

#### Temporal response fitting

**Figure 8:**
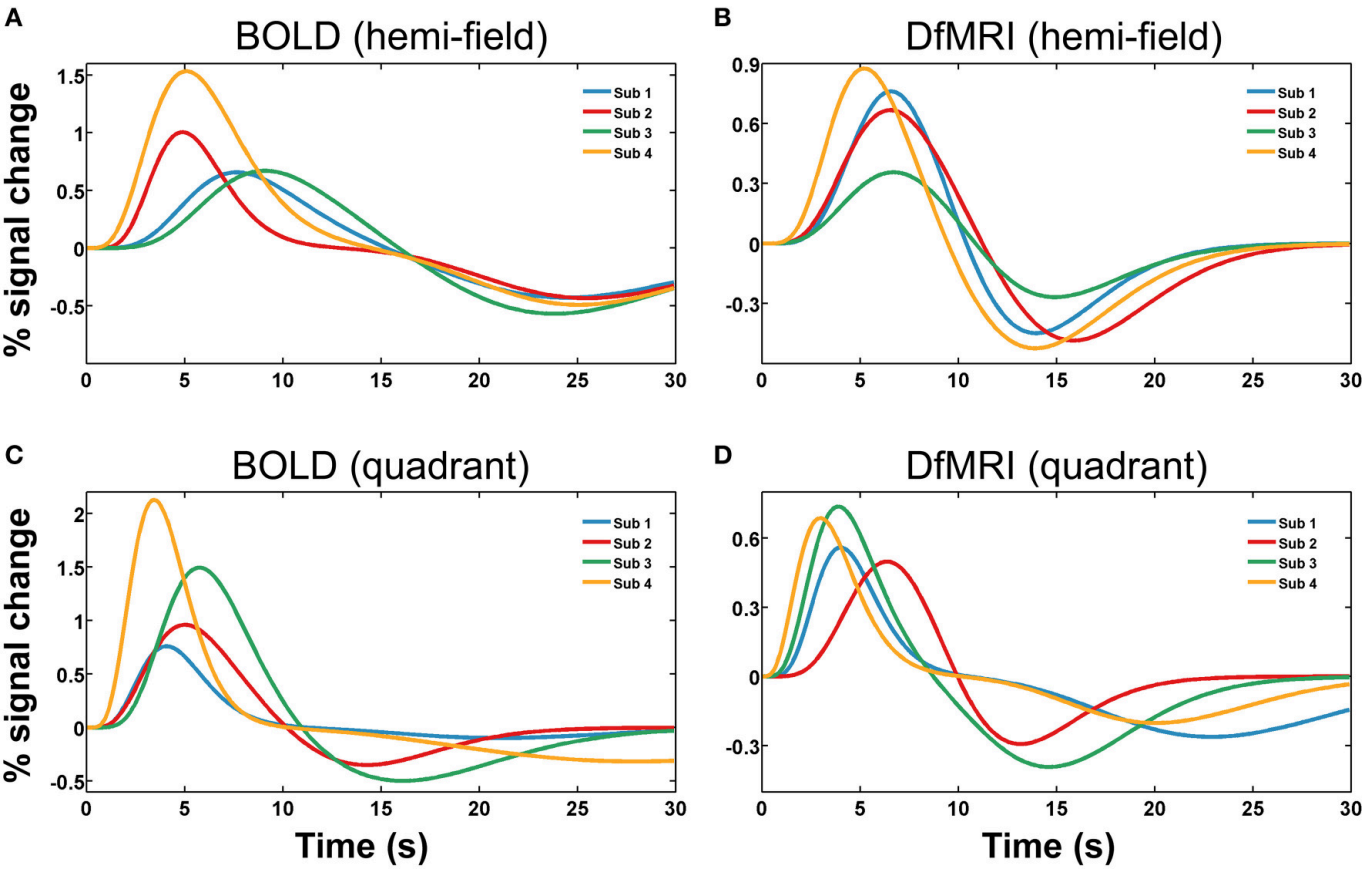
**Representative hemodynamic and diffusion response functions shown for four subjects (Sub 1, Sub 2, Sub 3, Sub 4), for four experimental conditions**. Panels show **(A)** BOLD_hemi_, **(B)** DfMRI_hemi_, **(C)** BOLD_quadrant_, and **(D)** DfMRI_quadrant_.

Runs of data with parameters exceeding one standard deviation from the group mean were excluded as outliers. The total number of runs removed from the fitting analysis as outliers was 20 (20%) for the DfMRI_quadrant_ condition, 18 (18%) for DfMRI_hemi_, 3 (15%) for BOLD_quadrant_, and 5 (25%) for BOLD_hemi_. The repeated-measures Friedman's ANOVA performed on the RSS between the estimated responses and the raw data confirmed that there was no significant difference between conditions in terms of fitting error, $\chi_{\left(3\right)}^{2}$ = 5.9, *P* = 0.12. A repeated measures two-way ANOVA performed on the parameters of the estimated response functions indicated a significant interaction between experimental condition and parameter, *F*_(9, 72)_ = 14.9, *P* < 0.0005. Four follow-up one-way ANOVA analyses with *post-hoc* paired comparisons showed that the experimental conditions differed significantly in terms of response width [*F*_(3, 24)_ = 11.1, *P* < 0.0005] and AUC [*F*_(3, 24)_ = 44.7, *P* < 0.0005]. For the *post-hoc* paired comparisons of width, all between-sequence comparisons reached significance (*P* < 0.05) however both BOLD and DfMRI did not show any difference between quadrant and hemi conditions. In terms of AUC, all the *post-hoc* comparisons reached significance (all *P*s < 0.01) except for the comparison between DfMRI_quadrant_ and DfMRI_hemi_ (*P* = 0.55). The ANOVA performed on TTP approached significance [*F*_(3, 24)_ = 2.7, *P* = 0.06], with the data showing a large difference between the TTP of the DfMRI_quadrant_ condition relative to the other three conditions. Because of this, paired comparisons were performed. These showed that the TTP of the DfMRI_quadrant_ condition was significantly shorter than the other three conditions (all *P*s < 0.04). The DfMRI_hemi_, BOLD_quadrant_ and BOLD_hemi_ conditions, however, did not differ in their TTP. There was no difference between the four conditions in terms of onset delay (*P* = 0.21). The parameters of the estimated temporal response functions averaged across participants are displayed in Table 2. Standard deviations are shown in parentheses. Representative response functions from four subjects are shown in Figure 8 for all four conditions. As demonstrated by Figure 8, there was a high degree of inter-subject variance in the shape of the estimated response functions.

**Table 2:** **Parameters of the averaged fitted response functions**.

**Condition**	**Time-to-peak**	**Width**	**Delay**	**AUC**
DfMRIquadrant	4.9 (±1.3)	4.0 (±0.68)	2.0 (±0.66)	1.9 (±0.38)
DfMRIhemi	5.8 (±0.83)	4.6 (±0.63)	2.4 (±0.63)	2.0 (±0.29)
BOLDquadrant	5.9 (±1.6)	5.7 (±1.4)	2.2 (±0.68)	4.7 (±1.3)
BOLDhemi	6.3 (±1.9)	6.7 (±2.1)	2.0 (±0.71)	5.5 (±1.5)

## Discussion

Spatial accuracy of the measured fMRI response to the locus of neural activity is critical for the accurate interpretation of brain activation maps. The BOLD contrast is inherently removed from the source of neural activity due to its reliance on vascular changes. Here we compared BOLD to a contrast sensitized to changes in water diffusion to determine the influence of BOLD and non-BOLD contributions to the diffusion signal. If DfMRI activation is more reliant on non-BOLD sources, it was anticipated that its activation profile would be more coherent with the known functional properties of the early visual cortex. Overall, DfMRI activation demonstrated some resemblance to a contrast source closer to the presumed underlying neural activity than the standard BOLD technique, however similarities between DfMRI and BOLD were identified. The significance of these findings will be discussed in greater detail.

It is established that the early visual cortex represents retinal stimulation in a point-by-point manner (Engel et al., 1997; Wandell and Winawer, 2011). These well-defined characteristics of the early visual cortex were reflected in the DfMRI activation patterns for the analysis on spatial extent. The significant positive relationship between hemi-field and quadrant activation patterns for DfMRI but not BOLD at the individual level indicated greater consistency between subjects. The finding of activation patterns highly consistent across subjects and localized to the presumed region of neural activity means that DfMRI may be of particular benefit to studies employing low subject numbers. The utility of this novel method may also be apparent when precise functional localization is required across subjects. However, when considering the finding of reduced between-subject variability reported here, the small sample size of the current study must be acknowledged. A larger sample size would provide a more accurate description of between-subject variance. Furthermore, the low sample size may affect the reproducibility of the current findings. With a larger sample size of 21, Aso et al. (2013) also reported reduced variability in the response magnitude obtained for DfMRI compared to BOLD, in line with the results reported here. Despite this, further research validating these findings of reduced across-subject variability would greatly benefit the continued use of DfMRI, and determine the reproducibility of the findings presented here.

One caveat is that for individual activation maps, spatial smoothing may be required, as the unsmoothed cluster sizes obtained for DfMRI were very small at a corrected threshold. The use of smoothing is likely to cause partial volume effects and negatively impact on the spatial specificity of the response. Overall, the low SNR of DfMRI is a vital concern that limits its practical implementation, both in the large amounts of data required to detect signal and the use of spatial smoothing. This may limit the subject populations that can undergo a study including DfMRI, and its ability to image at high spatial resolutions. For instance, given the low SNR of DfMRI, it would currently be unfeasible to differentiate DfMRI and BOLD profiles in different cortical layers of visual cortex *in vivo*. Such a study would benefit DfMRI by resolving the cortical layer of origin of the signal. Further concerns about the sensitivity of DfMRI to neuronal activity have been raised, based on recent work (Bai et al., 2016) in an *ex vivo* model which allowed simultaneous fluorescence imaging to monitor neuronal activity directly and MR imaging (Bai et al., 2015). In organotypic rodent cortical cultures, the diffusion fMRI signal was modulated by prolonged neuronal depolarization induced pharmacologically but not by spontaneous neuronal activity. The authors questioned the sensitivity of DfMRI to detect normal neuronal activity. It is clear from these results that further research to determine the biological basis of the non-BOLD aspects of the diffusion fMRI signal, in addition to research addressing its low sensitivity, is warranted.

The largest percent signal change was obtained here was found for the BOLD hemi-field condition. The positive relationship between BOLD signal amplitude and neural activity has been well-validated (Logothetis et al., 2001; Heeger and Ress, 2002), with an increase in BOLD signal corresponding to increases in cerebral blood flow, cerebral blood volume and vascular oxygenation levels related to cerebral metabolic rate of oxygen (Kim and Ogawa, 2012). The relationship between BOLD signal amplitude and area of cortical activation is less clear, although results reported by prior studies may suggest a link. For example, studies investigating linearity of spatial summation of BOLD response within the visual cortex have demonstrated a positive relationship between extent of stimuli and BOLD response. These studies addressed whether spatial summation is linear by determining whether the response to a larger visual stimulus can be predicted by the sum of responses to its fractionated components. Despite conflicting results of linear (Hansen et al., 2004) and subadditive (Kay et al., 2013) spatial summation, these studies reported larger BOLD amplitude to the stimulus occupying the greater portion of the visual field, in keeping with the BOLD results reported here. DfMRI, although demonstrating smaller signal changes relative to BOLD, was consistent with this previous work as amplitudes were slightly larger for the hemi-field relative to the quadrant condition.

The finding that DfMRI and BOLD signal changes did not correlate may be indicative of differing inter-individual variance between sequence types, suggesting that non-BOLD contributions are evident in DfMRI activation. Moreover, it was found here that BOLD signal amplitude significantly correlated with age, while DfMRI signal did not. This is in line with research demonstrating BOLD signal decreases with age, which appear to be associated with underlying vascular alterations (D'Esposito et al., 2003; Lu et al., 2011; Liu et al., 2013). While further research with larger sample size is necessary to support the findings reported here, it can be speculated that age-related vascular alterations may have contributed to our finding of a negative correlation between age and BOLD signal change.

In their work defining the diffusion response function, Aso et al. (2009) provided a model where the early portion of the diffusion response represented non-vascular sources, while the later component was dependent on the BOLD effect. In the present work characterizing the temporal response profiles, both DfMRI conditions differed significantly from BOLD in the width and area under the curve, with the diffusion response being narrower and with a faster offset. There was no difference between DfMRI and BOLD found in terms of onset delay, which is in contrast to the findings of Le Bihan et al. (2006), where a sharp increase in DfMRI signal from stimulus onset was reported. This may be due to the differences in acquisition parameters, as we implemented a slightly longer TR than this prior work. Interestingly, we report here a significantly shorter time-to-peak of the DfMRI_quadrant_ response, however a temporal precedence was not evident for the DfMRI_hemi_ condition. These findings may highlight sensitivity of the DfMRI signal to its BOLD contributions. It could be argued that the increased area of neural involvement in the hemi relative to the quadrant condition systematically induced an increased vascular response, thus demonstrating DfMRI sensitivity to neurovascular coupling. Future research comparing responses across conditions known to modulate the degree of neural activity would help to resolve this issue, and determine whether gradually increasing neural involvement systematically increases vascular contributions to DfMRI.

In the current study we compared the activation profiles of the diffusion response to BOLD using a paradigm that elicits a spatial summation effect in V1. We used gradient-echo (GE) BOLD fMRI as this is the gold-standard BOLD sequence, however spin-echo (SE) BOLD fMRI has been shown to reduce large vein contributions and localize signal to the smaller capillaries (Zhao et al., 2004). A comparison between DfMRI and SE BOLD in terms of spatial localization would benefit our understanding of the vascular compartments contributing to the diffusion signal. Previous research directly comparing SE BOLD and DfMRI has found that the DhRF- employed in the current study to model the diffusion response—is a more accurate description of the diffusion data, while the canonical hemodynamic response function (HRF) better fits the SE BOLD data (Aso et al., 2009). Overall, the HRF was estimated to contribute 26% of the total DfMRI signal. This suggests that SE BOLD and DfMRI are largely characterized by divergent temporal profiles. Further work examining the spatial properties of SE BOLD and DfMRI may advance our understanding of the diffusion signal source.

There are important considerations associated with the methodology of the present study. These include the known field asymmetry in visual processing, the influence of voluntary eye movements, the definition of V1 and the use of independent BOLD and DfMRI sequences. In regards to field asymmetry, the processing difference between the lower and the upper visual field is well-documented (Portin et al., 1999; Hagenbeek and Van Strien, 2002; Abrams et al., 2012; Hagler, 2014). Perceptual performance has shown biases toward the lower visual field relative to the upper visual field (Thomas and Elias, 2011), which may be due to asymmetries in the density of retinal cells (Curcio et al., 1990). We ran statistical analyses to determine whether such an asymmetry was evident in our data, by calculating percent signal change in voxels preferentially responding to lower visual field stimulation and comparing this to voxels responding to upper visual field stimulation. Our paired comparisons failed to reach significance, indicating that neither our DfMRI nor BOLD results were sensitive to visual field asymmetries. However, further work with larger sample size and stimuli that selectively target the lower and upper visual fields are warranted to validate the present findings.

Voluntary eye movements can change the visual field and the spatial location of the neural response to the field stimulation. In the present results, we observed activation in the ipsilateral cortex for both the BOLD smoothed and unsmoothed group analyses. However, we only found ipsilateral activation in the smoothed DfMRI images. While the ipsilateral activation may indicate participant gaze shift, the lack of it in the DfMRI unsmoothed analyses means that we cannot rule out an influence of spatial smoothing.

To characterize spatial specificity in the primary visual cortex, the use of retinotopic mapping to delineate the early visual regions may provide localizing information. Retinotopic mapping is dependent on the delay between the periodic stimuli and the phase of the BOLD signal (Bordier et al., 2015). Here we used atlas-defined ROIs, however future work may consider the use of retinotopic mapping for subject-specific ROI delineation.

A final consideration regarding methodology implemented here was the comparison of DfMRI and BOLD using independent sequences. The sequences implemented were consistent with prior research detailing DfMRI (Aso et al., 2009, 2013; Williams et al., 2014, 2015), which characterize the signal using a separate EPI sequence sensitized to diffusion. This indirect comparison to signal obtained from a separate BOLD-weighted sequence limits the analyses that can be performed. Group random-effects analyses were performed on DfMRI and BOLD separately, due to differing amounts of noise between the sequences and the different number of time points collected. The use of independent sequences also raises the possibility of variance induced from the differences between the sequences; for instance, the DfMRI sequence differed from the BOLD in terms of acoustic noise. These limitations should be considered carefully in the interpretation of results. A recommendation for future research is to consider a sequence which allows for the simultaneous collection of DfMRI and BOLD within a single TR.

In summary, we have demonstrated here comparisons between activation patterns obtained with DfMRI and BOLD fMRI to visual field stimulation. The data indicated that the residual vascular component of the diffusion signal does not impact on its ability to provide activation patterns that are consistent across subjects and localized to the primary visual regions. On the contrary, we identified important limitations surrounding the utilization of DfMRI. The low tSNR meant that more DfMRI data had to be acquired, drastically increasing scan time. Spatial smoothing was essential for analyses performed on individual activation maps. The current study employed a small number of subjects and used atlas rather than individually-defined ROIs, which may limit the interpretation of these findings. The lack of temporal precedence for the experimental condition inducing the larger cortical response indicated that the vascular contamination may be present and exerts influence on the diffusion temporal profile. Interestingly, the current findings indicated that the BOLD aspect of the DfMRI signal may not static, but dependent on experimental factors that include the extent of visual stimulation. It is clear that the physiological mechanisms driving the non-vascular aspects of the DfMRI response need to be empirically determined, and optimal experimental design and analysis of DfMRI data would address the early onset and later BOLD contamination to the signal. Thus, while determining the source of the early diffusion signal change is instrumental, future research also needs to consider the utility and practical implementation of DfMRI.

## Author contributions

RW was responsible for conceiving, designing, acquiring, analyzing, interpreting, and drafting this work. DR, JH provided substantial intellectual input into the conception, design, data acquisition, analysis, and interpretation. Both DR and JH drafted and gave final approval of this work.

### Conflict of interest statement

The authors declare that the research was conducted in the absence of any commercial or financial relationships that could be construed as a potential conflict of interest.
